# Investigating the tendency to use COVID-19 vaccine booster dose in Iran

**DOI:** 10.1186/s12913-023-09788-8

**Published:** 2023-10-02

**Authors:** Mehrdad Askarian, AmirAli Rastegar Kazerooni, zahra Shayan, Parisa Karimzadeh, Mohammad Movahedi, Nahid Hatam

**Affiliations:** 1 Department of Community Medicine, School of Medicine, Health Behavior Science Research Center, Shiraz University of Medical Sciences, Shiraz, Iran; 2 Student Research Committee, Shiraz University of Medical Sciences, Shiraz, Iran; 3 Department of Biostatistics, school of medicine, shiraz university of medical sciences, shiraz, Iran; 4 Department of Health, West Chester University, West Chester, PA USA; 5 Institute of Health Policy, Management, and Evaluation, University of Toronto, Toronto, Canada; 6 Department of Community Medicine, School of Medicine, Shiraz University of Medical Sciences, Shiraz, Iran

**Keywords:** COVID-19 vaccination, Vaccine hesitancy, Vaccination willingness, Booster dose

## Abstract

**Introduction:**

Vaccine hesitancy is recognized as a significant public health threats, characterized by delays, refusals, or reluctance to accept vaccinations despite their availability. This study, aimed to investigate the willingness of Iranians to receive booster shots, refusal rate, and their preferred type of COVID-19 vaccine.

**Materials and methods:**

This cross-sectional study was conducted over a month from August 23 to September 22, 2022 using an online questionnaire distributed through WhatsApp and Telegram online communities. The questionnaire assessed participants’ intent to accept COVID-19 booster vaccination and had no exclusion criteria. Data analysis involved using SPSS version 16.0, with t-tests and chi-square tests used to assess the bivariate association of continuous and categorical variables. A multivariate logistic regression model was built to examine the association between Health Belief Model (HBM) tenets and COVID-19 vaccination intent. The Hosmer Lemeshow Goodness of Fit statistic was used to assess the model’s fit, with a p-value > 0.05 indicating a good fit.

**Results:**

The survey was disseminated to 1041 adults and the findings revealed that 82.5% of participants expressed a desire to receive the booster dose. Participants who intended to be vaccinated were generally older (46.4 ± 10.9), mostly female (53.3%), single (78.9%), had received a flu vaccine (45.8%). The findings indicated that the HBM items, including perception of COVID-19 disease, perceived benefits of COVID-19 vaccines, COVID-19 safety/cost concerns, preference of COVID-19 vaccine alternatives, and prosocial norms for COVID-19 vaccination, received higher scores among individuals intending to be vaccinated compared to vaccine-hesitant individuals, with statistical significance (p < 0.05). However, the “COVID-19 risk-reduction habits” item had a higher score but did not reach statistical significance (p = 0.167).

**Conclusion:**

Factors such as lack of trust in the effectiveness of the vaccine, trust in specific vaccine manufacturers, and concerns about side effects of COVID-19 vaccine are among the most important factors. These findings have implications for national vaccination policies, emphasizing the need for policymakers in the health sector to address these factors as vital considerations to ensure the continuity of vaccination as one of the most important strategies for controlling the pandemic.

## Introduction

Severe Acute Respiratory Syndrome Coronavirus 2 (SARS-CoV-2) emerged in December 2019, rapidly spreading across China and the world, leading to a pandemic [1]. By June 2022, Iran had reported almost 7.2 million confirmed cases of COVID-19 and 141,350 confirmed deaths [2]. Initial efforts to vaccinate against COVID-19 have saved millions of lives allowing communities to gradually reopen and resume pre-pandemic activities [3]. Numerous articles have demonstrated the safety and cost-effectiveness of the vaccines [4, 5]. However, vaccine hesitancy poses a significant challenge in achieving herd immunity among the population [3].

Vaccine hesitancy, characterized by delays, refusals, or reluctance to accept vaccinations despite their availabilities, has been recognized as a major public health threats. Multiple factors contribute to vaccine hesitancy, including communication and media influence, racial and cultural factors, gender, socioeconomic and political barriers, vaccination experience, and the design of vaccination programs. Concerns about vaccine safety, effectiveness, and perceived risks of vaccine-preventable diseases are among the primary reasons for avoiding booster doses [6].

A previous study investigated the global trend in vaccine confidence and identifying gender (Male), years of education, and belonging to minority religious groups as negative factors while confidence in vaccines, eagerness to seek information, and trust in healthcare providers are positive factors in vaccination uptake [7]. Moreover, a rapid systematic review encompassing 126 studies examined factors related to vaccination receptivity over time, showing people’s perception of outbreak severity, risk of infection, and the safety, and effectiveness of vaccines. Other contributing factors including previous flu vaccination [8–10], self and community risk perceptions due to COVID-19 [9, 11], and being a health professional [12] which increase the likelihood of vaccine uptake.

Despite the aforementioned factors contributing to vaccine hesitancy, vaccination campaigns have been initiated globally and as of November 17th, 2022, 4,976,582,832 people, or about 62% of the world’s population, have received two doses of the COVID-19 vaccine. In the same dataset, 69.62% of Iranians had been vaccinated, which falls far below the desired percentage for achieving immunity against the pandemic [13]. It is crucial to consider the potential for RNA virus mutations, as they can impact the vaccine’s effectiveness against another strains of COVID-19. Vaccines approved for one strain may not be as effective against new mutations [14]. Therefore, due to the low vaccination rate alongside the emergence of new variants, the consumption of booster shots appears inevitable. Boosters of the COVID-19 vaccine can help increase or restore coverage against viruses that may have decreased over the time [15].

Multiple studies have addressed vaccine acceptance for primary vaccination, but the worldwide increase in COVID-19 variants and the decline in vaccine effectiveness have underscored the importance of booster injections. This study, aimed to investigate the willingness of Iranians to receive booster shots, refusal rate, and their preferred type of COVID-19 vaccine.

## Methods

### Design

In this cross-sectional study we employed an online questionnaire to assess the intention of individuals to accept COVID-19 booster vaccine.

### Data collection

The survey was distributed to a convenience sample via social media platforms, including WhatsApp and Telegram, over a period of over a month from August 23 to September 22, 2022. Survey participation was voluntary; no incentives were provided for completion. The study excluded individuals under the age of 18.

The minimum sample size was calculated as 390 based on a confidence level of 95%, a proportion of 50% and precision of 0.05. using the formula:

N = Z^2^ P (1 - P) / d^2^

Participants were presented with the survey’s objective and an online consent form upon accessing the survey homepage, those who agreed to participate could proceed by clicking the “Next” button at the bottom, while those who chose not to participate could terminate the survey. The data collection process ensured participant anonymity.

### Tools

The questionnaire consisted of three main sections. The first section collected demographic information including: age, gender, Job, marital status, pregnancy status, current location, highest level of education, work status, and whether the respondent was a health care worker (HCW). For HCWs, additional questions were included to determine if they worked in a hospital that admitted COVID-19 patients and if they direct exposure to COVID-19 patients. This section also gathered information about the history of chronic disease related to COVID-19 severity (such as diabetes mellitus, hypertension, lung diseases, renal diseases, cardiovascular diseases, and corticosteroids use), history of COVID-19 infection among respondents, history of COVID-19 disease among friends, colleagues, or family members, and history of flu vaccination, and number of doses of COVID-19 vaccine doses received.

The second section, titled “Health Belief Model (HBM) Domains,“ consisted of 26 items that had undergone factor analysis in a previous study [16].

These items were classified into six factors: which included perceptions of COVID-19 disease (three items), perceived benefits of vaccination (four items), concerns about COVID-19 vaccine safety and cost (four items), preferences for COVID-19 vaccine alternatives (four items), prosocial norms for COVID-19 vaccination (eight items), and COVID-19 risk-reduction habits (three items). Participants rated their responses to each item on a 5-point Likert scale ranging from ‘Strongly disagree’ (score: 1) to ‘Strongly agree’ (score: 5).

The third section focused on the primary outcome measure, which was the participants’ intention to receive a COVID-19 booster vaccine. This section contained one major item which asked the participants to rate their agreement with the statement “I will get the COVID-19 booster vaccine as soon as it is accessible” on a 5-point Likert scale ranging from one (strongly disagree) to five (strongly agree). For analysis, the scale was transformed into binary values of ‘Yes’ indicating ‘Strongly agree’ and ‘Agree’ answers and ‘No’ indicating the other responses. Additionally, participants were asked to indicate their preferred type of vaccine: foreign vaccine based on the country of manufacturing, a domestic vaccine, or no preference.

This study received approval from the institutional review board and ethics committee at Shiraz University of Sciences (SUMS) with the ethical code IR.SUMS.MED.REC.1401.041 and grant number 25,282.

### Data analysis

The collected data were analyzed using SPSS version 16.0 (IBM, Armonk, NY, USA). Descriptive statistics, including means and standard deviations, were calculated for scale items, while frequencies of responses for dichotomous variables. To examine the association between continuous variables and COVID-19 vaccination intent, T-tests were conducted. Chi-square tests were used to assess the bivariate association between of categorical variables and COVID-19 vaccination intent. To further explore the factors associated with COVID-19 vaccination intent, a multivariable logistic regression model was built using a forward stepwise process. The model aimed to examine the association between the Health Belief Model tenets and COVID-19 vaccination intent. The Hosmer Lemeshow Goodness of Fit statistic was used to assess the model’s fit to the data, with a p-value greater than 0.05 indicating a good fit. All sociodemographic variables and the six factors derived from the HBM were considered as potential candidates for model building. The significance level for variable selection in the regression model was set at p = 0.05.

## Results

### Participant characteristic

A total of 995 individuals completed the survey, out of 1041 adults who received it, resulting in a response rate of 95.6%. The mean age of respondents was 46 years (standard deviation [SD] of 11 years) ranging from 18 to 77 years. The majority of respondents were female (55.7%) and single (77.6%) (Table 1). Among the participants, 207 (20.8%) reported having at least one of the assessed chronic diseases, and 375 (37.7%) individuals reported being diagnosed with COVID-19 disease at least once during the pandemic. When asked about their intention to receive the COVID-19 vaccine, 821 participants (82.5%) responded with “agree”. Compared to those who did not agree or were unsure about receiving the vaccine, those who agreed to be vaccinated were, on average, older (p = 0.006), and a higher proportion were male (p = 0.001), single (p = 0.032) and had received a flu vaccine (p < 0.001).

**Table 1:** Sociodemographic and health characteristics of individuals who did and did not intend to receive COVID-19 vaccination

		Intent to receive COVID-19 vaccination		
		agree n (%) N = 821	disagree n (%) N = 174	p-value
**Age, mean (SD** ^**+**^ **)**		46.4 (10.9)	43.9 (10.3)	0.006^*^
**Gender**	Male	383 (46.7)	58 (33.3)	p < 0.001^**^
	Female	438 (53.3)	116 (66.7)	
**Marital status**	Married	151 (18.0)	40 (23.0)	0.032^**^
	Single	648 (79.0)	124 (71.3)	
	Others	22 (3.0)	10 (5.7)	
**Education**	Diploma	68 (8.0)	16 (9.2)	0.224^**^
	Bachelorette	260 (32.0)	65 (37.3)	
	Master	155 (19.9)	36 (20.7)	
	Doctorate	338 (41.1)	57 (32.8)	
**Healthcare worker**	Yes	399 (48.6)	80 (46.0)	0.529^**^
	No	422 (51.4)	94 (54.0)	
**History of chronic disease** ^**++**^	Yes	175 (21.3)	32 (18.4)	0.388^**^
	No	646 (78.7)	142 (81.6)	
**History of COVID-19 disease**	Yes	527 (64.2)	121 (69.5)	0.179^**^
	No	294 (35.8)	53 (30.5)	
**Family history of COVID-19 disease**	Yes	775 (94.4)	162 (93.1)	0.508^**^
	No	46 (5.6)	12 (6.9)	
**Received a flu vaccine**	Yes	376 (45.8)	54 (31.0)	P < 0.001^**^
	No	445 (54.2)	120 (69.0)	

^+^ SD: Standard deviation

^++^ Chronic diseases were any of the following diseases: diabetes mellitus, hypertension, lung disease, renal disease, cardiovascular disease, and corticosteroid consumption

^*^independent T-Test is used

^**^chi-square test is used

Except for the “risk-reduction habits” item, all other items showed significant between the two groups, with higher scores observed among those who agreed to receive the COVID-19 vaccine (Table 2).

**Table 2:** The score of the items by intent to receive COVID-19 vaccination

Items	Intent to receive COVID-19 vaccination		
	agree N = 821 mean (SD)	disagree N = 174 mean (SD)	p-value^*^
**Prosocial norms**	3.5 (0.95)	2.9 (0.58)	P < 0.001
**Perceived benefits of COVID-19 vaccination**	4.4 (0.89)	4.1 (0.77)	P < 0.001
**COVID-19 risk-reduction habits**	4.4 (0.69)	4.3 (0.75)	0.167
**COVID-19 vaccine safety/cost concerns**	3.4 (0.80)	2.9 (0.75)	P < 0.001
**Preference for COVID-19 vaccine alternatives**	4.1 (0.71)	3.9 (0.64)	P < 0.001
**Perceptions of COVID-19**	3.9 (0.64)	3.7 (0.59)	0.003

^*^ independent T-Test is used

### Multivariable logistic regression

All variables including sociodemographic factors and HBM tenets were included in the logistic regression analysis (Table 3). Gender and receiving a flu vaccine were found to be significantly associated with vaccination intent in the multivariable analysis. The odds of intending to receive a vaccination among males were 98% more than females (aOR = 1.98, 95% CI = 1.37, 2.86). Additionally, individuals who had received a flu vaccine had 59% higher odds of intending to receive a COVID-19 vaccination compared to those who had not received a flu vaccine (aOR = 1.59, 95% CI = 1.01, 2.30). Notably, the adjusted odds of intending to receive a COVID-19 vaccination increased by approximately 2.3 and 1.7 times for each one-point increase in the score of the scales assessing prosocial norms for COVID-19 vaccination and safety concerns about COVID-19 vaccine, (aOR = 2.23, 95% CI = 1.75, 2.83 and aOR = 1.67, 95% CI = 1.32, 2.11, respectively).

**Table 3:** Forward Stepwise Logistic Regression Analysis for Variables Predicting COVID-19 Vaccination Intent

Predictor variable		Adjusted Logistic regression		Un-adjusted Logistic regression	
		**OR (95% CI)**	P-value	**OR (95% CI)**	P-value
**Age**				1.02 (1.01, 1.04)	0.006
**Sex**	Male	1.98 (1.37, 2.86)	p < 0.001	1.75 (1.24, 2.47)	0.001
	Female	1		1.00	
**Marital status**	Married	1		1.00	
	Single			1.72 (0.75, 3.92)	0.199
	Others			2.38 (1.10, 5.14)	0.028
**Education**	diploma	1		1.00	
	bachelor			0.72 (0.39, 1.32)	0.287
	master			0.68 (0.46, 0.99)	0.048
	doctorate			0.73 (0.46, 1.15)	0.171
**Healthcare worker**	Yes			1.11 ( 0.80, 1.54)	0.53
	No	1		1.00	
**History of chronic disease** ^**+**^	Yes			1.20 (0.79, 1.83)	0.388
	No	1		1.00	
**History of COVID-19 disease**	Yes			0.79 (0.56, 1.12)	0.179
	No	1		1.00	
**Family history of COVID-19 disease**	Yes			0.51 (0.65, 2.41)	0.509
	No			1.00	
**Received a flu vaccine**	Yes	1.59 (1.09, 2.30)	0.015	1.88 (1.32, 2.66)	p < 0.001
	No	1		1.00	
**Prosocial norms**				2.10 (1.75, 2.54)	p < 0.001
**Perceived benefits of COVID-19 vaccination**		0.70 (0.56, 0.89)	0.003	1.38 (1.16, 1.64)	p < 0.001
**COVID-19 risk-reduction habits**				1.17 (0.94, 1.46)	0.168
**COVID-19 vaccine safety/cost concerns**		1.66 (1.32, 2.11)	p < 0.001	1.96 (1.58, 2.42)	p < 0.001
**Preference for COVID-19 vaccine alternatives**				1.56 (1.25, 1.95)	p < 0.001
**Perceptions of COVID-19**				1.47 (1.14, 1.89)	0.003

## Discussion

Vaccination is one of the most cost-effective strategies for preventing diseases. However, vaccine hesitancy which refers to the reluctance or refusal to vaccinate despite the availability of vaccines, poses significant threat to the progress made in combating vaccine-preventable diseases [17]. Therefore, it is crucial to examine individuals’ willingness to receive vaccinations and identify the factors that contribute to it. As the factors can change over the time [18], we conducted this study to investigate the inclination towards receiving a booster dose of the COVID-19 vaccine in Iran.

Our study revealed that out of 995 participants, 821 individuals (82.5%) expressed a desire to receive the booster dose. Among the participants who agreed A majority were older, male, single, and had received the flu vaccine.

Logistic regression analysis demonstrated that the odds of intending to receive the booster dose were 98% higher among males compared to females, and 59% higher among those who had received the flu vaccine compared to those who had not. furthermore, higher scores in prosocial norms and safety concerns about COVID-19 vaccination were associated with a greater intention to receive booster dose. However, there was a reverse correlation between the “perceived benefits” of COVID-19 vaccination and the intention to receive it.

Our study indicates a significant proportion (82.5%) of participants expressing a desire to receive the COVID-19 booster vaccine. This finding aligns with a study conducted by Suman Pal et al. among health care workers (HCWs) in the US, where only 7.9% of respondents expressed hesitancy towards receiving the booster dose. A similar study conducted in Japan reported a hesitancy rate of 6.7% [19], which is consistent with our survey [20]. However, another study conducted among adults in the US, including HCWs and non-HCWs, found that approximately 62% intended to take the booster dose, which the remaining individuals exhibited vaccine hesitancy [21].

Demographically, our study revealed that the mean age of participants willing to get vaccinated was higher compared to the three previous studies [19–21]. Several factors may contribute to this association; including a higher perceived risk of COVID-19 infection, serious illness, or the presence of comorbidities.

This study also identified gender as an important factor in vaccine intention, consistent with two other studies [19, 21].

Our findings indicate various that various components of the HBM, such as “perception of COVID-19 disease”, “Perceived benefits of COVID-19 vaccines”, “COVID-19 safety/cost concerns”, “Preference of COVID-19 vaccine alternatives”, “prosocial norms for COVID-19 vaccination” received higher scores among vaccine-intended individuals compared to vaccine-hesitant ones, with statistical significance. However, “COVID-19 risk-reduction habits” obtained a higher score without reaching statically significance. Given the multiple waves of pandemic in Iran along with a high number of deaths and hospitalizations, participants appeared to prioritize the issue more seriously.

Another noteworthy finding of our study was the association between “receiving flu vaccination” and the intention to receive COVID-19 vaccine booster. Previous studies have also shown that uptake of the flu vaccine contributes to the acceptance of the COVID-19 vaccine [22–24]. This can be explained by the fact that individuals who have previously received the flu vaccine and have observed its positive effects such as reduced duration of flu-like symptoms and mild side effects, tend to develop trust in vaccinations, leading to an increased intention to receive the COVID-19 vaccine.

Furthermore, “prosocial norms for COVID-19 vaccination” emerged as a significant contributing factor in our study (aOR = 2.23, 95% CI = 1.75, 2.83). A study conducted in Japan by Mikiko Tajiya et al. similarly highlighted the significant role of “social norms” in vaccine booster intention [19]. Social norms encompass factors such as influence from family, friends, media and physicians. Another study conducted in China, demonstrated that lower vaccine hesitancy was associated with a higher perceived importance of social media as a component of prosocial norms [25].

### Strengths and limitations

To best of our knowledge, this study represents the first investigation conducted to assess the intention to use a COVID-19 vaccine booster dose in Iran. Notably, our study includes a substantial population sample that participated in the survey, enhancing the generalizability of the findings. It is important to note that due to the cross-sectional nature of this study, the results can only establish an association. Thus, we strongly recommend the implementation of a cohort study to assess the intention to use a COVID-19 vaccine booster dose. This would provide valuable insights into the stability and durability of our findings, allowing us to determine if the intention to use a booster dose remains consistent over the time. A cohort study would enable a longitudinal assessment of individuals’ intentions, thereby enhancing our understanding of their willingness to receive a COVID-19 vaccine booster dose over an extended period. By investigating this important aspect, we can gain further confidence in the reliability and generalizability of our results.

## Conclusion

Firstly, our study revealed significant associations between male gender and previous flu vaccine uptake with the likelihood of receiving a COVID-19 vaccine booster. Secondly, our study demonstrated the significant impact of “prosocial norms in COVID-19 vaccination” and “safety concerns” on vaccine intention. This underscores the influence of social norms and individuals’ perception of safety in shaping their willingness to receive a vaccine booster dose.

Considering these findings, it is crucial for policy-makers to place emphasis on the continuation of vaccination efforts as one of the most essential strategies for controlling the pandemic. By prioritizing these aspects, policy-makers can effectively encourage vaccine uptake and ultimately combat the spread of the virus more effectively.

## Data Availability

All data generated or analyzed during this study are included in this published article.
